# Advanced oxidation protein products change biological behaviors of rat endometrial epithelial cells by activating ERK/P38 signaling pathways

**DOI:** 10.1242/bio.048876

**Published:** 2020-06-01

**Authors:** Jing Liu, Sixi Wen, Yanling Lin, Xiaoping Yang, Zebang Liu, Song Quan, Yali Song

**Affiliations:** 1 Center for Reproductive Medicine, Department of Obstetrics and Gynecology, Nanfang Hospital, Southern Medical University, Guangzhou 510515, People's Republic of China; 2 Center for Reproductive Medicine, Department of Obstetrics and Gynecology, Peking University Shenzhen Hospital, Shenzhen 518000, People's Republic of China; 3 College of Engineering, Huazhong Agricultural University, Wuhan 43000, People's Republic of China

**Keywords:** AOPPs, Endometrial epithelial cells, Biological behaviors, ERK, P38

## Abstract

Advanced oxidation protein products (AOPPs) are a family of oxidized protein compounds and could induce oxidative stress and inflammatory lesion in various cells. The accumulation of AOPPs was associated with female reproductive diseases such as polycystic ovary syndrome (PCOS), leiomyoma and endometriosis. However, the relationship between AOPPs and endometrial cells is unclear. To explore the effects of accumulated AOPPs on endometrial cells, we treated normal rat endometrial epithelial cells (rEECs) and endometriosis model rats with AOPPs. Primary rEECs were collected from 8-week-old female Wistar rats. Increasing the amount of AOPPs in the media of rEECs enhanced rEEC proliferation and migration, and inhibited apoptosis. Moreover, AOPPs triggered the production of reactive oxygen species and nitrite along with activated ERK and P38 signal and this, in turn, led to an upregulation of proliferation and migration. With the treatment of antioxidants or the inhibitors of ERK and P38, the above effects of AOPPs on rEECs were attenuated. Additionally, in an endometriosis rat model, a similar phenomenon was observed in that the growth of endometriotic implants were promoted by AOPPs and EECs were significantly increased. This study indicated that the accumulation of AOPPs could promote rEEC proliferation and migration through ERK and P38 signal both *in vivo* and *in vitro*.

This article has an associated First Person interview with the first author of the paper.

## Abstract (precis)

**Summary:** The research indicated that the accumulation of AOPPs could promote rat endometrial epithelial cells proliferation and migration through ERK and P38 signaling pathways both *in vivo* and *in vitro*.

## INTRODUCTION

Endometrial epithelial cells (EECs) are the major cell type in the endometrium. The normal morphology and function of EECs plays a vital role in maintaining female reproductive health. But in certain pathological conditions, the morphology of EECs is destroyed and its function is changed, such as excessive proliferation, invasion, epithelial-to-mesenchymal transition and fibrosis, which lead to the occurrence of reproductive diseases (Sun et al., 2019; You et al., 2019; An et al., 2017; Meng et al., 2019). There is a growing body of literature showing that the abnormal proliferation and migration of EECs is related to the progression of endometriosis (Meng et al., 2019).

Numerous factors have been implicated in the pathogenesis of EECs proliferation and migration: sex hormones – both estrogen and progesterone, reactive oxygen species (ROS), and transforming growth factor-β have been extensively characterized (Wang et al., 2020; Mohankumar et al., 2019). Recently, a family of oxidized protein compounds, named advanced oxidation protein products (AOPPs), have emerged as novel markers of ROS-mediated protein damage that are usually carried by plasma proteins (Meyer and Schmitt, 2000). Not only do AOPPs result from oxidative stress, but they also result in oxidative stress (Saeidnia and Abdollahi, 2013). Multiple studies have demonstrated that AOPPs play a vital role in various pathologies. AOPPs may accelerate renal fibrosis and atherosclerosis, and they may be detrimental to the progress of chronic kidney disease (Liu et al., 2006; Li et al., 2007; Cao et al., 2013). In postmenopausal women, AOPPs are negatively associated with a decreased bone mineral density, which increases an individual's risk of developing osteoporosis (Wu et al., 2015). Notably, the detrimental effects of AOPPs on the female reproductive system have received increasing attention. The level of serum AOPPs is significantly increased in women with polycystic ovarian syndrome (PCOS) (Moti et al., 2015). In patients with uterine leiomyoma, the serum level of AOPPs increases and the antioxidant capacity decreases (Santulli et al., 2013). In peritoneal fluid and follicular fluid of endometriosis, the level of AOPPs is also extraordinarily high (Santulli et al., 2015; Song et al., 2018). These observations suggest that AOPP accumulation has a close relationship with endometriosis, but it remains unknown whether AOPPs affect biological characteristics of EECs.

Mitogen activated protein kinase (MAPK) signaling pathway has been found to play a pivotal role in various cell behaviors including proliferation, migration, apoptosis and so on (Mormile and Vittori, 2013). In i*n vitro* research, the increased ROS level in EECs was associated with increased proliferative capacity (Ngô et al., 2009). With increasing O_2_^−^ level, ERK and P38 level in EECs present in an increasing tendency (Yoshino et al., 2004; Ngô et al., 2009). This research suggests that ROS affects EECs behavior via activating the MAPK signaling pathway. However, whether the MAPK signaling pathway mediates AOPPs-associated biological properties in EECs is unclear.

Hence, this study was designed to determine the contribution of AOPPs in changing biological behaviors of rat endometrial epithelial cells (rEECs) both *in vivo* and *in vitro*. Our data show that the accumulation of AOPPs prompts rEEC proliferation and migration. It is mainly mediated by activating ERK and P38 signaling pathways.

## RESULTS

### Purification of rEECs

**Fig. 1.**
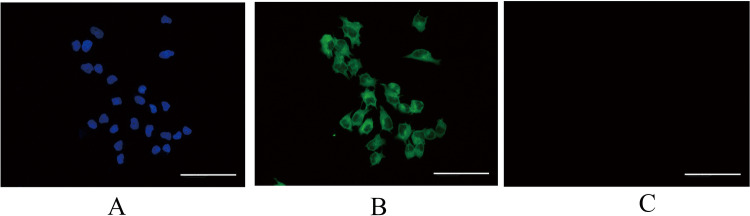
**Images of extracted primary rEECs.** (A) Nuclei of epithelia cells stained with 4,6-diamidino-2-pheylindole (DAPI; blue). (B) Epithelial cells stained with fluorescein isothiocyanate labeled anti-cytokeratin antibodies (green). (C) Epithelial cells stained with fluorescein isothiocyanate labeled anti-vimentin antibodies (no fluorescein). Original magnification ×400. rEECs, rat endometrial epithelial cells.

From an endometrium specimen, rEECs were extracted and endometrial stromal cells were discarded. The cell nucleus of extracted cells showed blue fluorescein for DAPI (Fig. 1A). The rEECs showed green fluorescein after being stained with fluorescein isothiocyanate anti-cytokeratin antibodies and showed negative staining for vimentin antibodies, which was in agreement with a previous study (Yamagami et al., 2014) (Fig. 1B,C).

### Increase in extracellular AOPPs was sufficient to trigger proliferation and migration, and block apoptosis of rEECs

**Fig. 2.**
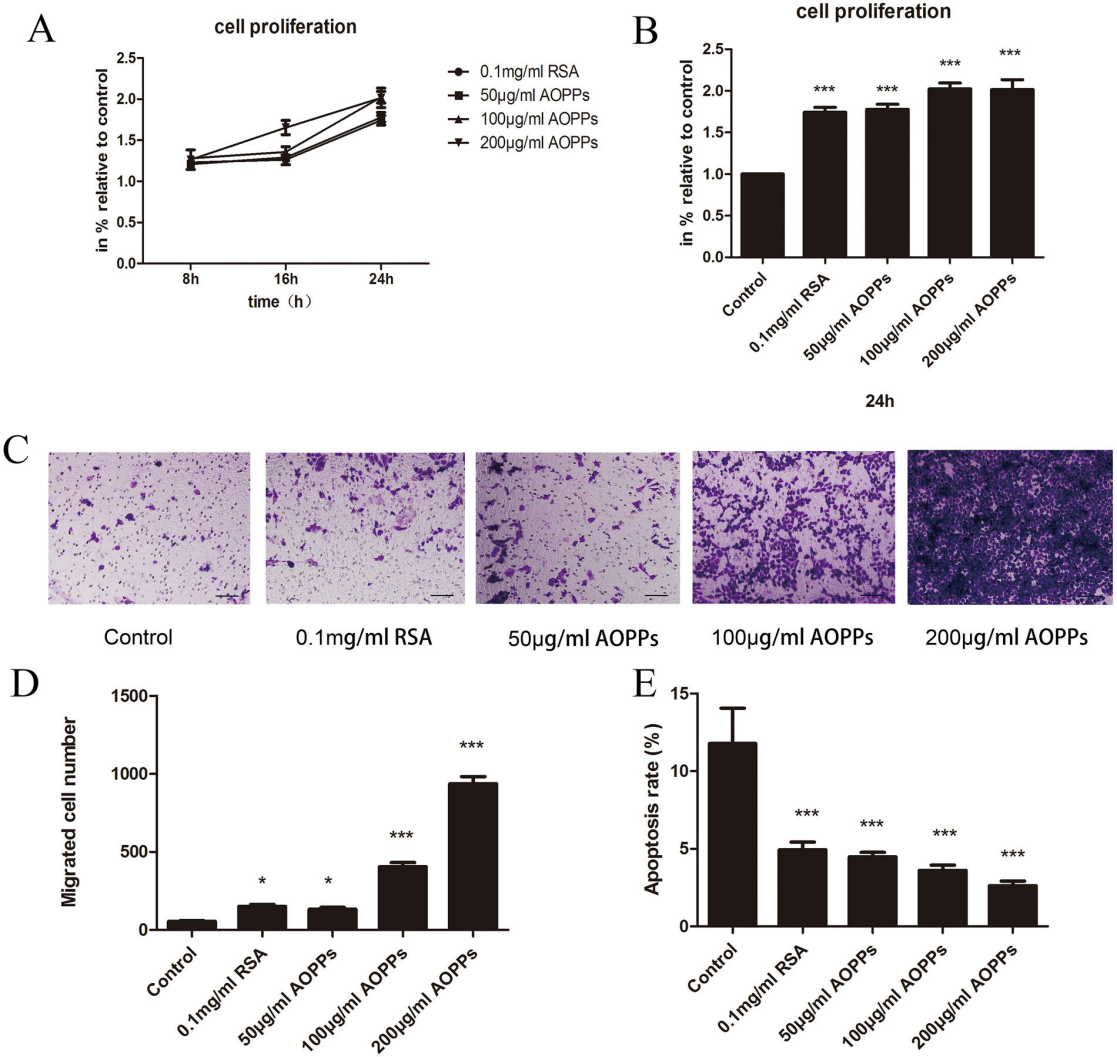
**AOPP induced proliferation and migration in rEECs.** Cells treated with medium alone served as controls. (A) Proliferative rEECs significantly increased in a dose- and time-dependent manner. (B) Proliferation of rEECs treated with indicated concentrations of AOPPs or native RSA for 24 h. (C) Migrated rEECs with different substrates (medium, RSA, AOPPs) for 24 h on the lower surface of the transwell membranes. Original magnification ×200. (D) Average number of migrated rEECs with different substrates (medium, RSA, AOPPs) for 24 h. (E) Apoptotic rEECs significantly decreased after cells were treated with indicated concentrations of AOPPs or native RSA for 24 h. Data are expressed as mean±s.d. of three independent experiments. *P<0.05, ***P<0.001 versus control. AOPPs, advanced oxidation protein products; RSA, rat serum albumin.

To explore the cells’ biological changes brought about by AOPPs, cultured rEECs were incubated with indicted concentration of AOPP-modified rat serum albumin (RSA) for indicated time (see the Materials and Methods). Exposure of rEECs to AOPPs induced a dose- and time-dependent increase in proliferation as assessed by MTT (Fig. 2A). After cells were treated with 100 μg/ml AOPPs for 24 h, the proliferation rate was 2.02±0.07 and significantly higher than the control group (Fig. 2B). However, there was no significant difference in proliferation between the 100 μg/ml AOPPs group and 200 μg/ml AOPPs group (*P*=0.909), when the duration of treatment was up to 24 h. In addition, if the culture time extended through 36 h or 48 h, primary rEECs viability decreased remarkably. Thus, 100 μg/ml was selected as the concentration of AOPPs and 24 h was selected as the duration for further experiments.

Meanwhile, migration of rEECs showed a dose-dependent increase. In each view, the average number of migrated rEECs in the 100 μg/ml AOPPs group and the 200 μg/ml AOPPs group was significantly higher than the control group (Fig. 2C,D).

Correspondingly, rEECs apoptosis was distinctly inhibited. Decreased apoptosis was observed in all rEECs incubated with AOPPs, but there was no significant difference among AOPPs-treatment groups (Fig. 2E).

### AOPPs challenge increased ROS and nitrite generation *in vitro*

**Fig. 3.**
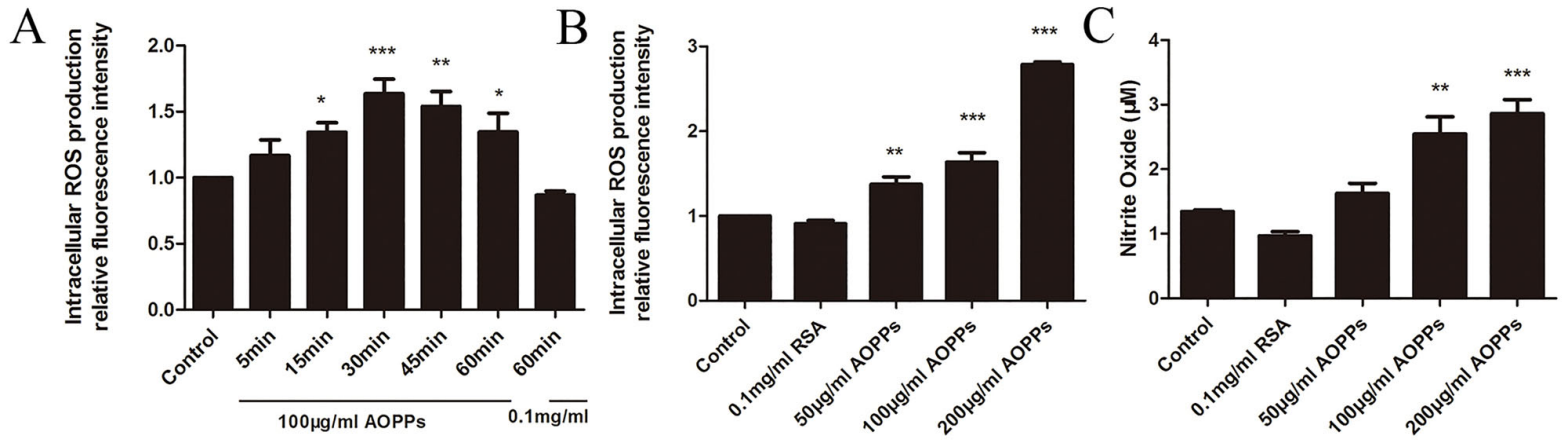
**AOPP challenge induced ROS and nitrite generation in rEECs.** Cells treated with medium alone served as controls. (A) ROS production in rEECs treated with 100 μg/ml AOPPs or native RSA within 1 h. (B) ROS production in rEECs treated with indicated concentrations of AOPPs or native RSA for 30 min. (C) Nitrite production in rEECs treated with indicated concentrations of AOPPs or native RSA for 24 h. Data are expressed as mean±s.d. of three independent experiments. *P<0.05, **P<0.01, ***P<0.001 versus control. AOPPs, advanced oxidation protein products; RSA, rat serum albumin; ROS, reactive oxygen species; rEECs, rat endometrial epithelial cells.

To explore the underlying mechanism of AOPP-induced biological changes in rEEC properties, ROS and nitrite were examined in rEECs culture solution. ROS production presented time-dependent and dose-dependent increases in AOPP-treated rEECs. After cells were treated with 100 μg/ml AOPPs for 30 min, the relative fluorescence intensity in rEECs reached peak value and was significantly higher compared with cells incubated with medium alone (Fig. 3A). At 30 min, the relative fluorescence intensity was significantly increased with an increase in AOPPs concentration (Fig. 3B).

Similarly, nitrite production was induced to increase in AOPP-treated rEECs. Especially, when the concentration of AOPPs attained 100μg/ml, the nitrite production presented significantly higher (Fig. 3C).

### AOPP-induced changes of cells properties were associated with increased phosphorylation of ERK and P38

**Fig. 4.**
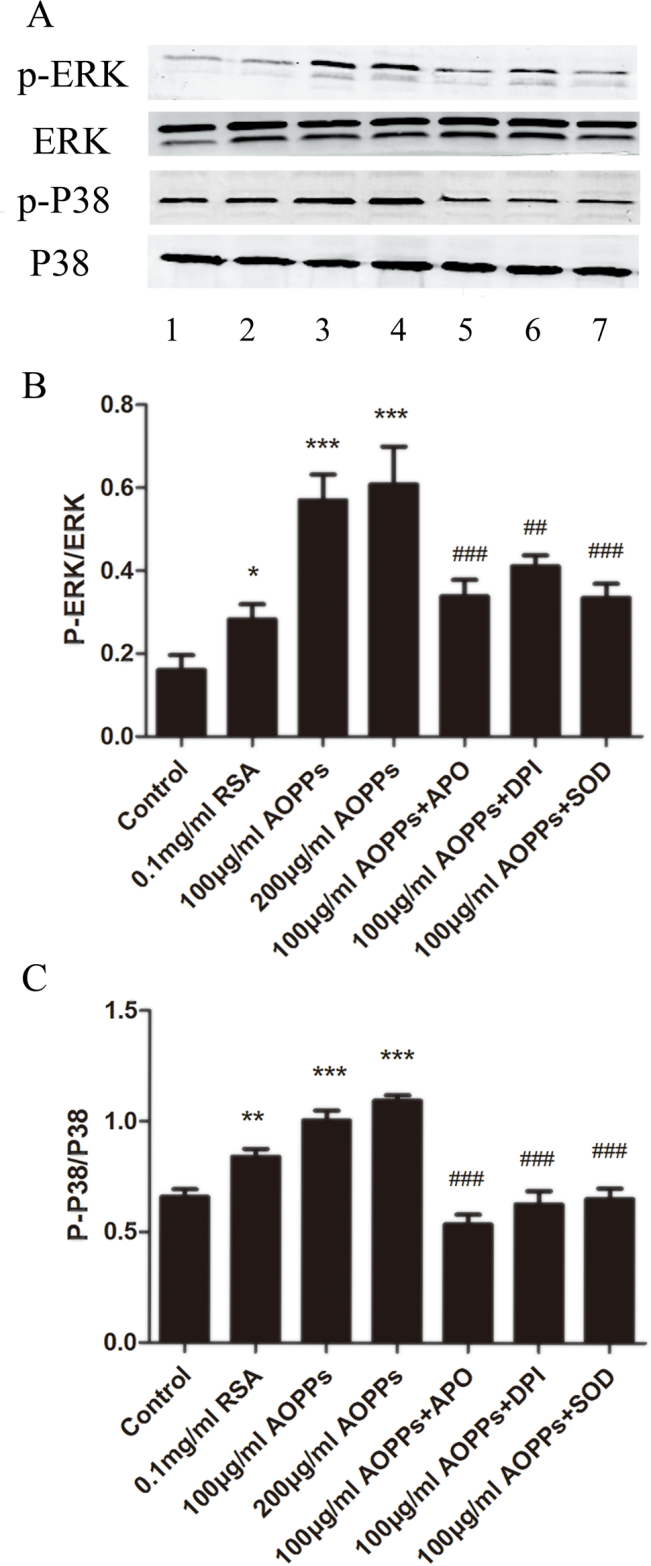
**AOPP challenge increased p-ERK and p-P38 expression by activating oxidative stress.** Cells treated with medium alone served as controls. (A) The representative western blots of p-ERK, ERK, p-P38 and P38 in rEECs with different treatments. (B) AOPP-induced upregulation in p-ERK could be blocked by pretreatment of rEECs with APO, DPI and SOD. (C) AOPPs-induced upregulation in p-P38 could be blocked by pretreatment of rEECs with APO, DPI and SOD. Data are expressed as mean±s.d. of three independent experiments. *P<0.05, **P<0.01, ***P<0.001 versus control; ^##^*P*<0.005, ^###^*P*<0.001 versus AOPPs. AOPPs, advanced oxidation protein products; RSA, rat serum albumin; rEECs, rat endometrial epithelial cells; p-ERK, phosphorylation-ERK; p-P38, phosphorylation-P38; APO, apocynin; DPI, diphenyleneiodonium; SOD, superoxide dismutase.

To examine the potential mediators of AOPP-triggered biological changes in rEECs, we analyzed the abundance of phosphorylation-ERK (p-ERK) and phosphorylation-P38 (p-P38) with western blot analysis. AOPP challenge increased the expression of p-ERK and p-P38 in rEECs. The p-ERK/ERK and p-P38/P38 in AOPP-treated rEECs were significantly increased compared with those incubated with medium alone or native RSA (Fig. 4). These data suggest that AOPP-induced cell biological changes are associated with increased activity of ERK and P38 signaling pathways. Enhanced expression of p-ERK and p-P38 could be completely blocked by the antioxidant apocynin (APO), diphenyleneiodonium (DPI) and superoxide dismutase (SOD). In rEECs pre-incubated with APO, DPI and SOD, p-ERK/ERK decreased to 59%, 72% and 59%, respectively, compared to those treated with only AOPPs, and p-P38/P38 decreased to 53%, 62% and 65%, respectively, compared to those treated with only AOPPs, suggesting that AOPP-induced activation of ERK and P38 signaling pathways is mainly mediated by oxidative stress (Fig. 4).

**Fig. 5.**
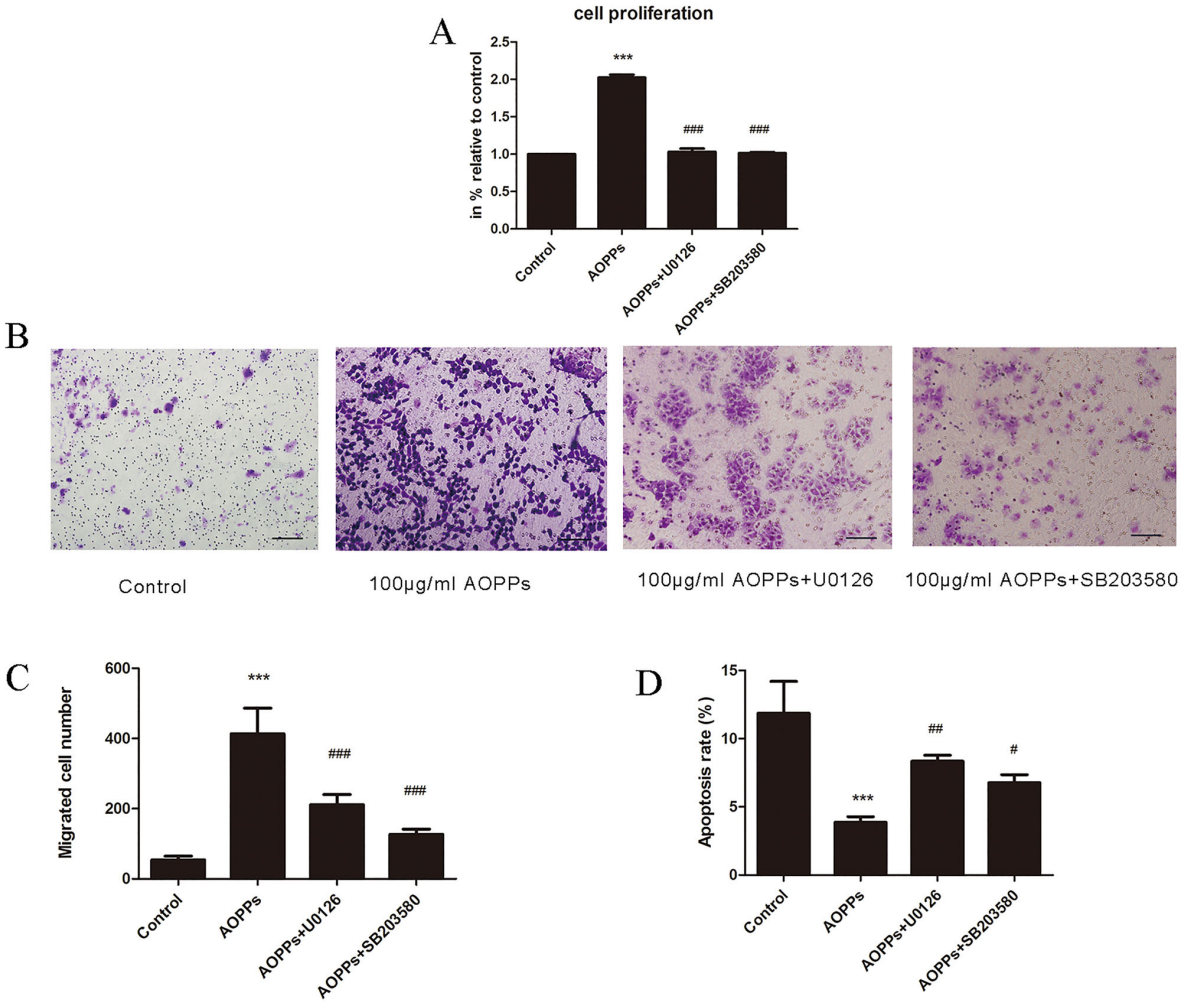
**Specific blockers for ERK and P38 attenuated AOPP-induced rEEC proliferation and migration.** Cells treated with medium alone served as controls. (A) Proliferation of rEECs pre-incubated with the inhibitors of ERK (U0126) or P38 (SB203580) for 2 h and followed by 100 μg/ml AOPPs treatment for 24 h. (B) Migrated rEECs with different treatments on the lower surface of the transwell membranes. Original magnification ×200. (C) Average number of migrated rEECs pre-incubated with the inhibitors of ERK (U0126) or P38 (SB203580) for 2 h and followed by 100 μg/ml AOPPs treatment for 24 h. (D) Apoptosis of rEECs pre-incubated with the inhibitors of ERK (U0126) or P38 (SB203580) for 2 h and followed by 100 μg/ml AOPPs treatment for 24 h. Data are expressed as mean±s.d. of three independent experiments. ***P<0.001 versus control; ^#^*P*<0.05, ^##^*P*<0.005, ^###^*P*<0.001 versus AOPPs. AOPPs, advanced oxidation protein products; RSA, rat serum albumin; rEECs, rat endometrial epithelial cells; APO, apocynin; DPI, diphenyleneiodonium; SOD, superoxide dismutase.

Furthermore, to verify the association between ERK/P38 signaling pathways and rEECs property changes triggered by AOPPs, rEECs were pre-treated for 2 h with inhibitors of signaling pathways – specific blocker for ERK (U0126) and specific blocker for P38 (SB203580). Then, the cells were incubated with AOPPs for 24 h. The rEEC proliferation and migration levels triggered by AOPPs were decreased and apoptosis showed increased levels compared with cells treated with AOPPs alone, suggesting that AOPP-triggered changes of rEEC biological properties are mediated through ERK and P38 signaling pathways (Fig. 5A–D).

### AOPP-induced changes of cell properties were associated with increased ROS and nitrite generation

**Fig. 6.**
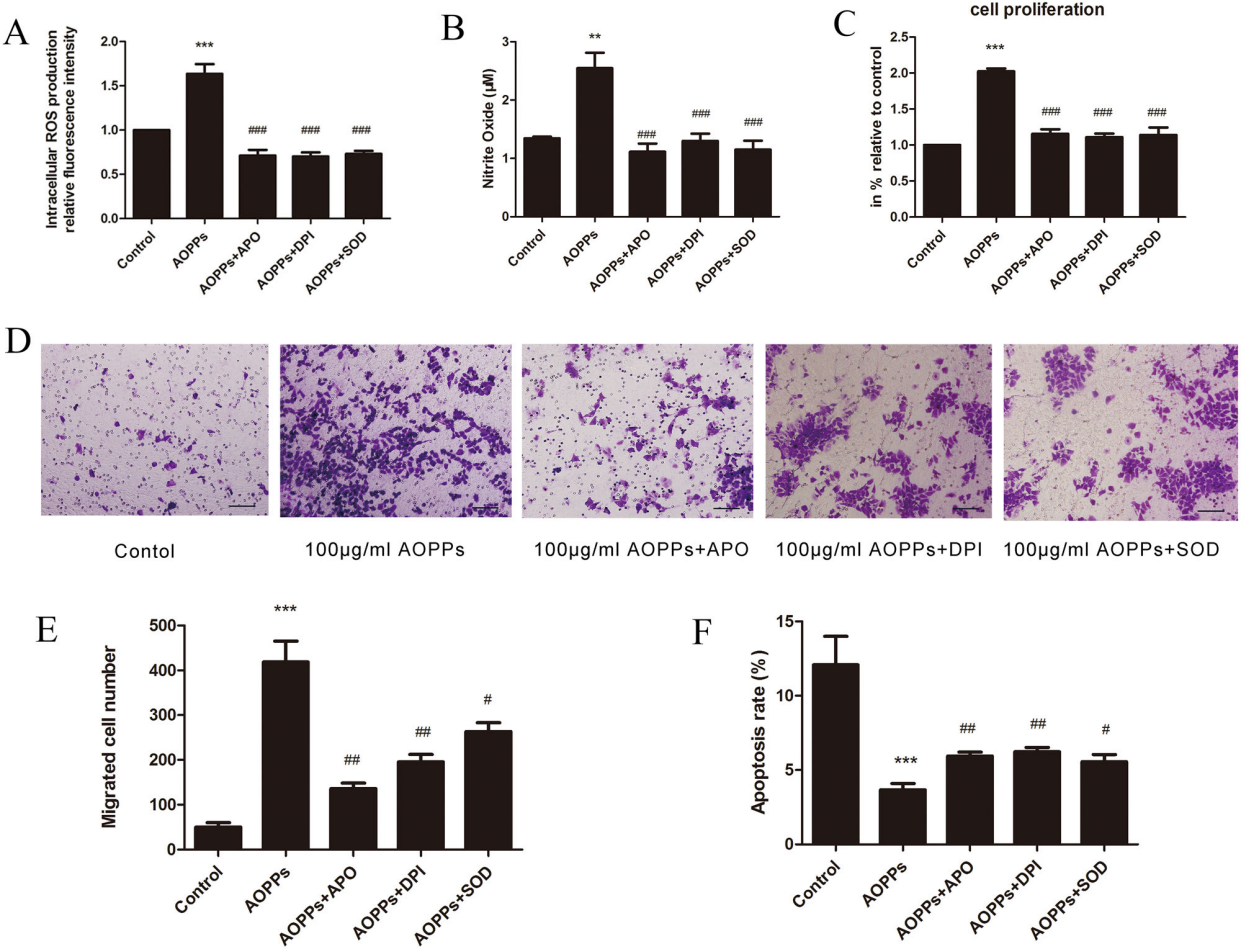
**Specific blockers for ERK and P38 attenuated AOPP-induced rEEC proliferation and migration.** Cells treated with medium alone served as controls. (A) ROS production in rEECs pre-treated with antioxidants (APO, DPI, SOD) for 1 h and followed by 100 μg/ml AOPPs treatment for 24 h. (B) Nitrite production of rEECs pre-treated with antioxidants (APO, DPI, SOD) for 1 h and followed by 100 μg/ml AOPPs treatment for 24 h. (C) Proliferation of rEECs pre-incubated with antioxidants (APO, DPI, SOD) for 1 h and followed by 100 μg/ml AOPPs treatment for 24 h. (D) Migrated rEECs with different treatments on the lower surface of the transwell membranes. Original magnification ×200. (E) Average number of migrated rEECs pre-incubated with antioxidants (APO, DPI, SOD) for 1 h and followed by 100 μg/ml AOPPs treatment for 24 h. Data are expressed as mean±s.d. of three independent experiments. (F) Apoptosis of rEECs pre-incubated with antioxidants (APO, DPI, SOD) for 1 h and followed by 100 μg/ml AOPPs treatment for 24 h. **P<0.005, ***P<0.001 versus control; ^#^*P*<0.05, ^##^*P*<0.005, ^###^*P*<0.001 versus AOPPs. AOPPs, advanced oxidation protein products; RSA, rat serum albumin; ROS, reactive oxygen species; rEECs, rat endometrial epithelial cells; APO, apocynin; DPI, diphenyleneiodonium; SOD, superoxide dismutase.

To further clarify the effect of oxidative stress on AOPP-induced biological changes in rEECs properties, the rEECs were pre-incubated for 1 h with APO, DPI and SOD. The rEECs were then treated with AOPPs for 24 h. AOPP-triggered ROS and nitrite generation was almost completely blocked by pre-incubating rEECs with APO, DPI and SOD, suggesting that AOPP challenge triggered O_2_^−^ generation in rEECs (Fig. 6A,B). Meanwhile, rEECs proliferation and migration induced by AOPPs were both significantly suppressed by those three inhibitors, and rEECs apoptosis was distinctly increased after pre-treatment with inhibitors, suggesting that AOPP-triggered cell biological changes are mainly dependent on O_2_^−^ generation (Fig. 6C–F).

### AOPPs induced the development of ectopic implants

**Fig. 7.**
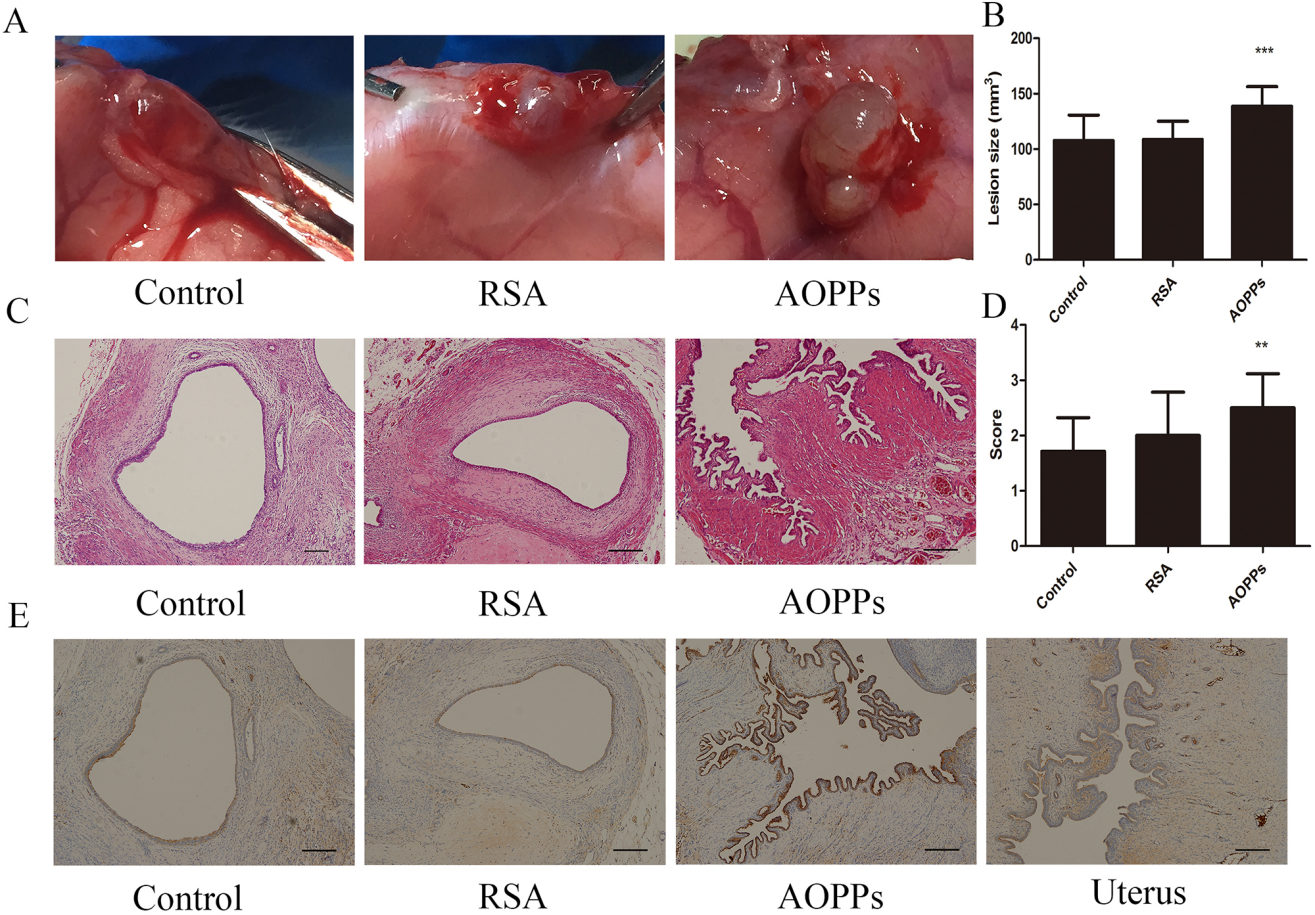
**Rats model of endometriotic implants.** (A) Macroscopic implants of rats treated successively with PBS, RSA (50 mg/kg/day), AOPPs (50 mg/kg/day). (B) Volume of active lesions in rats treated successively with PBS, RSA (50 mg/kg/day), AOPPs (50 mg/kg/day). (C) Histology of active lesions in rats treated with PBS, 50 mg/kg/day RSA, 50 mg/kg/day AOPPs. Original magnification ×100. (D) Pathology score of active lesions in rats treated successively with PBS, RSA (50 mg/kg/day), AOPPs (50 mg/kg/day). (E) Immunohistochemistry staining of keratin in active lesions of rats treated with PBS, RSA (50 mg/kg/day), AOPPs (50 mg/kg/day) and normal uterine of rats. Original magnification ×100. **P<0.005, ***P<0.001 versus control. AOPPs, advanced oxidation protein products; RSA, rat serum albumin.

To confirm *in vitro* findings, 18 Wistar rats received endometriotic implants to build animal models and different treatments. Rats treated with PBS, RSA and AOPPs all showed active lesions with angiogenic and glandular organization (score 1.71±0.61 for PBS; score 2.00±0.78 for RSA; score 2.50±0.62 for AOPPs) (Fig. 7). The volume of implants in the control group and RSA group was significantly lower than that in the AOPPs group (control group versus AOPPs group: 107.78±22.97 mm^3^ versus 138.69±17.77 mm^3^, *P*<0.001; RSA group versus AOPPs group: 108.79±16.44 mm^3^ versus 138.69±17.77 mm^3^, *P*<0.001) (Fig. 7A–D). Pathology scores were both obviously different between the control group and the AOPPs group (*P*=0.007) (Fig. 7E–H). Whereas, there was not a significant difference in pathology score between the control group and the RSA group (Fig. 7E–H). Keratin was positively detected in the glandular epithelium (Fig. 7I–L). Thus, AOPPs can promote the proliferation and migration of endometrial epithelial cells in a rat animal model.

## DISCUSSION

AOPPs, as a class of potential gynecology pathogenic mediators and the multiple means by which they form in various disorders, have been recognized for their role in the female reproductive system. This study provided *in vivo* and *in vitro* evidence showing that stimulation of AOPPs triggered rEEC proliferation and migration, and suppressed apoptosis through inducing ROS and nitrite generation, and activating ERK and P38 signaling pathways. To the best of our knowledge, this is the first report exposing the effects of AOPPs on rEECs.

Endometrial cells could accomplish implantation outside the uterus via migration, adhesion, invasion and vascularization (Nisolle et al., 2000). Notably, the abnormal biological behaviors of EECs are closely associated with the occurrence of endometriosis, adenomyosis and endometrial cancer (Streuli et al., 2015; Matsuzaki and Darcha, 2014). However, the underlying mechanism of mediating biological behaviors of rEECs has not been completely understood. Several factors have been recognized to induce endometrial cell proliferation and migration; ROS, sex hormones, transforming growth factor-β, interleukin-32 and tumor necrosis factorα (TNF α) have been extensively characterized (Ngô et al., 2009; Kocbek et al., 2016; Lee et al., 2018; Wang et al., 2020; Mohankumar et al., 2019). In this study, we found that accumulation of AOPPs in the media of rEECs enhanced rEEC proliferation and migration, and inhibited apoptosis, suggesting AOPPs may have a close relationship with endometrial properties change. Additionally, in our endometriosis rat model, a similar phenomenon was observed in that the growth of endometriotic implants was promoted by AOPPs and EECs were significantly increased.

We next explored the underlying mechanism. To address the pathogenesis, we first analyzed ROS and nitrites, the two key products of oxidative stress in rEECs. We found that the effect of AOPPs on rEECs was dependent on triggering ROS and nitrite generation. After rEECs were treated with AOPPs, the levels of ROS and nitrite in rEECs increased. In addition, ROS production was positively correlated with AOPP levels. When the concentration of AOPPs attained 100μg/ml, the incentive effect on nitrite production in rEECs also presented obvious. In accordance with previous studies, the increase of ROS and nitrite was accompanied by AOPP accumulation (Hébert-Schuster et al., 2012; Garibaldi et al., 2017).

Other studies have revealed MAPK signaling pathway is closely associated with proliferation and migration of either tumor cells or normal cells (Huo et al., 2015). To further investigate the underlying mechanism of AOPP-induced biological characteristics of EECs, we tested p-ERK and p-P38 of the MAPK signaling pathway. Our results showed that p-ERK and p-P38 were both activated by AOPPs in a dose-dependent tendency, which was in the same manner as cell behaviors. Moreover, the AOPP-triggered biological disorders of rEECs could be almost completely blocked by U0126 (the inhibitor of ERK) and SB203580 (the inhibitor of P38), suggesting that the increased proliferation and migration, and decreased apoptosis are all dependent on activated ERK and P38.

It is noteworthy that oxidative stress has been verified to be one of the triggers of the MAPK signaling pathway, and both of them act as the accelerant for cell proliferation and migration (Chen et al., 2013; Huo et al., 2015). Thus, we next verified whether the effect of AOPPs on EECs resulted from activated oxidative stress via the MAPK signaling pathway. In our study, APO, DPI and SOD were used as pre-treatment for rEECs to study further. APO and DPI are the inhibitors of NADPH oxidase, and SOD is the scavenger of O^2−^ (Vejrazka et al., 2005; Chen et al., 2013; Wang et al., 2015). Many studies have stated that these antioxidants could relieve damage caused by oxidative stress and block the progression of diseases associated with oxidative stress (Meyer and Schmitt, 2000; Tawfik et al., 2009; Zheng et al., 2013; Cao et al., 2013). Similarly, in our research, the response of rEECs to AOPPs was weakened by antioxidants. Interestingly, with the inhibition of ROS and nitrite brought on by antioxidants, the expression of p-ERK and p-P38 decreased, and the effect of AOPPs on biological behaviors of rEECs were all attenuated. Taken together, our results suggest that AOPPs might promote the production of ROS and nitrite in rEECs, and overabundant ROS and nitrite products activate the MAPK/ERK and MAPK/P38 signaling pathways to improve rEEC proliferation and migration.

To further distinguish the changes of rEEC biological properties brought on by either AOPPs or RSA, a group of rEECs treated with RSA was necessary. During this treatment, proliferation and migration increased, and p-ERK and p-P38 were also activated, but the effects brought by AOPPs were more obvious than those by RSA. Additionally, RSA treatment did not increase the production of ROS and nitrite. It demonstrated that the effect of RSA on rEECs has no correlation with oxidative stress. AOPPs, not native albumin, induced the generation of ROS and nitrite, alterations of cell behavior and dysregulation of ERK and P38 signaling pathways, suggesting that the oxidative stress-associated triggering is due to AOPPs, not a property of RSA.

Nevertheless, the effects of AOPPs on more rEECs properties, such as invasion, angiogenesis, metaplasia and reproductive function, are still unclear. Further studies should focus on exploring more changes in rEEC properties that have resulted from AOPPs; whether AOPPs damage the reproductive function of endometrial cells and what the potential mechanism is.

In conclusion, we showed that AOPP challenge could promote rEEC proliferation and migration, and suppress apoptosis via activated ERK and P38 signaling pathways, both *in vivo* and *in vitro*. This research might be highly significant because it not only reveals new insight into the mechanism of biological property changes in rEECs, but also helps to investigate the pathogenesis of diseases associated with endometrium.

## MATERIALS AND METHODS

### Ethical approval

All studies involving animals were performed after receiving the approval of the Animal Care and Use Committee (IACUC) of Nanfang Hospital, Southern Medical University, China.

### Cells isolation and culture

Considering the differences among human samples, primary rEECs were collected from the endometrium of 8-week-old female Wistar rats (weight 200–250 g, Southern Medical University Animal Experiment Center) to maintain consistency. Eligible Wistar rats were anesthetized with intraperitoneal injection of 4% chloralic hydrate (Hatch, Shanghai, China). A laparotomy was performed on the ventral midline and endometrium specimens were scraped from incised uterus. Specimens were rinsed and minced into small pieces, digested and separated according to the methods of Ngô et al. (2009). rEECs were retained on 40 μm aperture sieves and plated onto Primaria flasks (Corning, USA). Dulbecco's modified Eagle's medium (DMEM) (Gibco Invitrogen, France) with 10% fetal calf serum (Gibco Invitrogen, France) were applied to culture cells. Purification of rEECs were confirmed by staining with a 1:1000 dilution of fluorescein isothiocyanate labeled anti-cytokeratin and anti-vimentin antibodies (Cell Signaling Technology, USA). The Olympus fluorescent microscope (Germany) was used to analyze fluorescence and capture pictures.

### AOPPs albumin preparation

AOPPs were prepared *in vitro* by incubation of RSA (Sigma-Aldrich, USA) with 40 mM NaClO (Guangzhou Chemical Reagent Factory, Guangzhou, China) in the absence of free carbohydrates/amino acid/lipids to exclude the formation of advanced glycation end product-like structures as description (Guo et al., 2008). Prepared samples were dialyzed against phosphate-buffered saline (PBS) to remove hypochlorous acid and passed through a Detoxi-Gel column (Pierce, USA) to remove contaminated endotoxin. The level of endotoxin in the samples was measured with the amebocyte lysate assay kit (Sigma-Aldrich, USA) and were found to be below 0.025 EU/ml.

AOPPs content in the preparation, which was determined by the absorbance of the mixture of samples and acetic, was 66.1±4.7 mmol/mg protein. The components of advanced glycation end products were undetected in the prepared samples.

### Assessment of rEEC proliferation

Proliferation experiments were performed with the MTT assay. The rEECs were incubated at a density of 1×10^4^ cells/well in a 96-well plate. Cells were incubated in a 96-well plate with DMEM with 10% fetal calf serum for 24 h, and then the culture medium was replaced by only DMEM and cells were continuously incubated for 4 h. Subsequently, rEECs were treated according to description in Table 1. For measuring proliferation rate, 20 μl MTT solution (5 mg/ml in normal saline) was added to each well, and then the plates were kept at 37°C for 4 h. The absorbance was measured on an enzyme-linked immunosorbent assay (ELISA) reader (SpectraMax, USA) at a wavelength of 492 nm. Data were presented in optical density units.

**Table 1.**
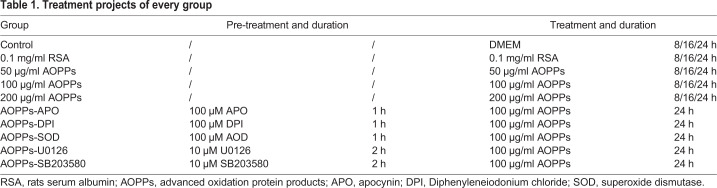
**Treatment projects of every group**

### Assessment of rEEC apoptosis

The rEECs were incubated at a density of 3.5×10^5^ cells/well in six-well plates and treated as described in Table 1. According to the manufacturer's instructions (Sigma-Aldrich, USA), the rEEC suspension was incubated with either fluorescein isothiocyanate (FITC)-conjugated AV alone, Propidium Iodide (PI) alone, or a combination of both. Apoptotic analysis was performed using flow cytometry (Becton Dickinson, USA), with excitation at 488 nm and emission at 530 nm (for cells labeled with AV) and 620 nm (for cells labeled with PI). Single labeling was used to gate and control for bleed throughout. The rEEC population was characterized on the basis of whether it was labeled with AV alone (apoptotic), PI alone (necrotic), neither AV nor PI (viable), or both AV and PI (late apoptotic).

### Assessment of rEEC migration

The evaluation of migration was performed with 24-well transwell chambers (8 mm, BD bio-coat-matrigel invasion chamber, Becton Dickinson, USA). After the above treatment, 200 μl of rEEC suspension was seeded into upper compartments at the density of 5×10^5^ cells/ml. The lower chambers were filled with DMEM containing 5% fetal calf serum. After 48 h, rEECs on the upper surface of the filter were removed by wiping with a cotton swab. Cells on the lower surface of the membrane were fixed in methanol for 20 min, stained with 0.1% Crystal Violet for 30 min and washed three times with PBS. After staining, migrated cells on the lower surface were visualized and photographed with an upright microscope (Olympus, Germany). For each group, migrated cell populations were enumerated in five random visual fields and the counts were averaged.

### Assessment of ROS generation

ROS generation was measured by using the peroxide-sensitive fluorescent probe 2,7-dichlorofluorescein diacetate (DCF-DA) (Sigma-Aldrich, USA). DCF-DA is converted by intracellular esterases into DCFH, which is oxidized into the highly fluorescent dichlorofluorescein (DCF) in the presence of a proper oxidant (Eruslanov and Kusmartsev, 2010). The rEECs were seeded into 96-well plates at the density of 1×10^4^ cells/well and cultured with DMEM containing 10% fetal calf serum for 24 h, and continuously starved for 4 h. After incubation in 10 μM DCF-DA for 30 min at 37°C, rEECs were treated separately as described in Table 1. After the medium was all replaced by PBS, the fluorescence intensity was assessed by the ELISA reader (SpectraMax, USA) with excitation/emission wavelength of 488/525 nm.

### Assessment of nitrite generation

Nitrite was measured with the Griess method by using corresponding commercial assay kits (Nanjing Jiancheng Bioengineering Institute, Nanjing, China). The rEECs were incubated at a density of 4×10^5^ cells/well in six-well plates and treated as described in Table 1. Supernatant was collected to assess nitrite production according to the manufacturer's instructions. Optical densities were measured at 550 nm.

### Western blot analysis

To measure the protein expression of related signaling pathways, rEECs (3.5×10^5^ per well) were seeded in six-well plates and treated as described in Table 1. Then rEECs were lysed for protein extraction according to the manufacturer's instructions (Beyotime Biotechnology, Shanghai, China), after which protein concentration was measured by using a BCA assay kit (Beyotime Biotechnology, Shanghai, China). The lysate containing equal protein was subjected to 8% SDS-PAGE for electrophoresis and transferred onto polyvinylidene fluoride (PVDF) membranes (Millipore, USA). Blots were blocked with 5% BSA in Tris Buffered Saline Tween (TBST) and incubated with the appropriate primary antibodies diluted 1:1000 to: mouse anti-ERK1/2 antibody (Cell Signaling Technology, USA), mouse anti-phosphorylantion-ERK1/2 antibody (Cell Signaling Technology), mouse anti-P38 antibody (Cell Signaling Technology), and mouse anti-phosphorylantion-P38 antibody (Cell Signaling Technology). Subsequently, the membranes were incubated with fluorogenic secondary antibodies at room temperature for 1 h. The blots were visualized and photographed with Odyssey system (LI-COR, USA).

### Animal model

18 female Wistar rats (weight 200–250 g, 8-weeks-old, Southern Medical University Animal Experiment Center) were used to build the animal model. Rats received humane care under the institutional guidelines. Estrous rats were anesthetized as described previously. A laparotomy was performed on the ventral midline and right uterine was incised. 5-mm^2^ fragments of endometrium were sutured to the bilateral parietal with 7/0 absorbable suture. The cutis was sutured with 4/0 nylon thread. Rats were randomized into three groups and received a daily intraperitoneal injection of vehicle (endotoxin-free PBS, PH7.4), native RSA (50 mg/kg/day), or AOPPs (50 mg/kg/day), respectively. On day 19 after implantation, rats were euthanized by cervical dislocation. Volume measurement of implants was calculated using the formula: *V*(mm^3^)=(length×width^2^)/2 (all in mm). Histological analysis was applied to ectopic implants. Tissue slides were stained with Hematoxylin and Eosin to score pathologically. Pathology scores were performed separately by three different pathologists according to the criterion described by Ngô et al. (2009). To confirm the persistence of endometrial epithelial cells, immunohistochemistry was applied to stain slides with 1:200 dilution of anti-cytokeratin (Cell Signaling Technology). All stained slides were observed and photographed under an upright microscope (Olympus).

### Statistical analysis

All results of studies are the means of independent triplicate experiments for each cellular population of each rat. In all experiments, the cells were plated at the same density. Data were expressed as mean±standard deviation (s.d.). The statistical significance of differences in mean values was analyzed with SPSS Statistics 20.0 (SPSS Inc., USA). One-way ANOVA test was applied to compare means. When the two-tailed *P*-value was <0.05, the differences were considered statistically significant (*P<0.05, **P<0.01, ***P<0.001).
